# Chasing Jenner's Vaccine: Revisiting *Cowpox Virus* Classification

**DOI:** 10.1371/journal.pone.0023086

**Published:** 2011-08-08

**Authors:** Darin S. Carroll, Ginny L. Emerson, Yu Li, Scott Sammons, Victoria Olson, Michael Frace, Yoshinori Nakazawa, Claus Peter Czerny, Morten Tryland, Jolanta Kolodziejek, Norbert Nowotny, Melissa Olsen-Rasmussen, Marina Khristova, Dhwani Govil, Kevin Karem, Inger K. Damon, Hermann Meyer

**Affiliations:** 1 Poxvirus and Rabies Branch, Centers for Disease Control and Prevention, Atlanta, Georgia, United States of America; 2 Biotechnology Core Facility Branch, Centers for Disease Control and Prevention, Atlanta, Georgia, United States of America; 3 Oak Ridge Institute for Science and Education, Oak Ridge, Tennessee, United States of America; 4 Division of Microbiology and Animal Hygiene, Department of Animal Sciences, Georg-August-University, Göttingen, Germany; 5 Department of Food Safety and Infection Biology, Section of Arctic Veterinary Medicine, Norwegian School of Veterinary Science, Tromsø, Norway; 6 Genøk, Center for Biosafety, The Science Park, Tromsø, Norway; 7 Zoonoses and Emerging Infections Group, Clinical Virology, Department of Pathobiology, University of Veterinary Medicine Vienna, Vienna, Austria; 8 Department of Microbiology and Immunology, Faculty of Medicine and Health Sciences, Sultan Qaboos University, Muscat, Oman; 9 Bundeswehr Institute of Microbiology, Munich, Germany; Univ. of Texas HSC at San Antonio, United States of America

## Abstract

*Cowpox virus* (CPXV) is described as the source of the first vaccine used to prevent the onset and spread of an infectious disease. It is one of the earliest described members of the genus *Orthopoxvirus*, which includes the viruses that cause smallpox and monkeypox in humans. Both the historic and current literature describe “cowpox” as a disease with a single etiologic agent. Genotypic data presented herein indicate that CPXV is not a single species, but a composite of several (up to 5) species that can infect cows, humans, and other animals. The practice of naming agents after the host in which the resultant disease manifests obfuscates the true taxonomic relationships of “cowpox” isolates. These data support the elevation of as many as four new species within the traditional “cowpox” group and suggest that both wild and modern vaccine strains of *Vaccinia virus* are most closely related to CPXV of continental Europe rather than the United Kingdom, the homeland of the vaccine.

## Introduction

*Cowpox virus* (CPXV) is one of the earliest described members of the genus *Orthopoxvirus* (OPV). Historically, researchers referred to the ailment known as cowpox and even suggested that it could provide immunity against smallpox [1]. However it was Jenner's publications in 1798 and 1799 which provided the first scientific description of vaccination by detailing the efficacy of CPXV “scarification” in inducing protective immunity against challenge with variola virus (VARV) [2], [3]. The common name “cowpox virus” refers to the association with pustular lesions on the teats of milking cows and historic zoonotic transmission of this disease to humans (milkers) through contact with infected cows. Human infections are generally mild and self-limiting with localized skin lesions healing after 3–4 weeks, however, systemic involvement and fatal outcome have been reported in immunocompromised individuals [4], [5].

CPXV is believed to be endemic to Eurasia, but cowpox-like illness has also been reported in Israel [6]. The last few decades have witnessed an increasing number of reports of human CPXV infection in Europe, as well as the infection of a broad range of domestic and zoological garden animals including; elephants, primates, cats, and pet rodents. Cowpox in cats was first described by Thomsett in 1978 [7]. The first zoonotic transmission of CPXV from cat to human was documented in 1985 from the Netherlands. CPXV genomes isolated from cats in England are closely related to those obtained from cows and their handlers in the same region [8], [9]. In addition, more than 30 outbreaks have been described in zoo animals between 1960 and 2010. Long-term ecological studies on CPXV indicate that, although the virus rarely has been isolated from them, wild rodents are the likely reservoir hosts of CPXV and infection rates vary seasonally [10], [11], [12]. Recent human cases have been primarily associated either with contact with pet rats or contact with infected cats (presumably infected by wild rodents), and not from contact with infected cattle.

Among *Orthopoxvirus* species, traditionally defined CPXV have the largest genomes averaging above 220 kbp, about 30 kbp larger than the average known *Vaccinia virus* (VACV) genome. Only the genome from *Horsepox virus*, a presumed “wild” VACV isolate, is larger than 200 kb (212633 bases). But like other OPVs roughly 30–40% of the CPXV genome encodes products that play important roles in virus pathogenesis and host range. Several CPXV open reading frames (ORFs) have nucleotide sequence similarity to immunomodulatory and host range function genes, while others interfere with host activation cascades [13].

Phenotypic features differentiating currently recognized CPXV from other OPV species are the presence of large eosinophilic A-type inclusion (ATI) bodies in the cytoplasm; the induction of 2–4 mm flattened, fairly rounded pocks with a red central hemorrhagic area on the chorioallantoic membrane (CAM) of embryonated eggs at 72 hours post inoculation; and large, indurated, hemorrhagic lesions on scarified rabbit skin. Restriction fragment length polymorphisms (RFLP) generated with *Hin*dIII, and to lesser extent *Bam*HI, *Xho*I and/or *Sma*I, and construction of physical genome maps, have been used to differentiate between CPXV strains. These methodologies revealed higher genotypic variation in German CPXV isolates than those from Scandinavia and the UK, which suggests the potential for geographically independent evolution of these viruses and perhaps differences in their rodent reservoir species [14].

Previous phylogenetic studies comprised of fewer viral isolates and based on more limited sets of proteins or nucleotide sequences from single genes have reported a “remarkable divergence” between CPXV strains, specifically CPXV Brighton Red and CPXV Grishak, and have suggested these two isolates could represent different species [15], [16]. In this study we include both a wider range of isolates and present phylogenetic and genomic analyses based on the whole genome from nine CPXV strains circulating in Europe as well as three genomes of CPXV from Great Britain, Moscow and Germany, sequenced previously (Brighton Red, GRI-90, GER 1991-3). This more expanded taxonomic and genotypic data set clearly suggests CPXV, as currently recognized, is a polyphyletic assemblage. Therefore the current CPXV taxonomic rank must be divided into multiple species which each represent a monophyletic lineage.

## Materials and Methods

Whole genome (nucleotide) sequences from 12 isolates identified as CPXV were utilized in this study. The locations and details relevant to these isolates are listed in Table 1. Three fully sequenced CPXV strains were obtained from GenBank: CPXV strain Brighton Red (CPXV BR; 224,499 bp, 235 ORFs, GenBank No: AF482758) originating from the hand of a milker in Great Britain in 1938; strain GRI-90 (223,666 bp, 214 ORFs, GenBank No: X94355) isolated in 1990 from a 4 year old girl in Moscow that had contact with a mole and CPXV strain OPV GER 1991-3 (228,250 bp, 219 ORFs, GenBank No: DQ 437593) isolated in 1991 from a human case in Germany. Whole genome sequences from nine of these isolates were generated for this study using Sanger sequencing methodologies as previously described [17]. These sequences were deposited in GenBank and accession numbers can be found in Table 1. MAFFT [18] was used to align the OPV coding region (VACV C23L-B29R) for the twelve CPXV isolates with the following additional genome sequences: *Variola virus* (L22579, Y16780), *Taterapox virus* (NC_008291), *Camelpox virus* (CMLV) (AY009089, NC_003391), M*onkeypox virus* (MPXV) (DQ011156, DQ011155), four vaccine (*Vaccinia virus*) strains (AY678276, M35027, AM501482, DQ377945) as well as three non-vaccine VACV strains including one strain from a buffalo from India, one from a horse in Mongolia (DQ792504), and one laboratory-obtained, Rabbitpox virus Utrect isolate (AY484669). Two *Ectromelia virus* isolates (NC_004105, see www.poxvirus.org for *Ectromelia virus* strain Naval) were included as outgroup taxa. After the complete alignment was obtained, all gapped columns were excluded leaving a datset with a length of 145204 bp.

**Table 1:** CPXV isolates.

Strain	Place of isolation	Year	Clinical description	Host	Reference	Accession #
CPXV_gri	Russia, Moscow	1990	Local lesions	human	Marennikova et al. 1996 Zh Mikrobiol 4:6–10	X 94355
CPXV_BR	United Kingdom, Brighton	1937	Local lesions	human	Downie 1939 British Journal of Experimental Pathology 2o:I58–176	NC 003663
CPXV_GER1991_3	Germany, Munich	1991	Local lesions	human	Meyer et al. 1999 Arch Virol 144:491–501	DQ 437593
CPXV_FIN2000_MAN	Finland, Tohmajärvi	2000	Generalized lesions	human	Pelkonen et al. 2003 EID 9:1458–61	HQ420893
CPXV_NOR1994_MAN	Norway, Bergen	1994	Local lesions	human	Tryland et al. 1998 Scand J Infect Dis 30:301–303	HQ420899
CPXV_GER1990_2	Germany, Bonn	1990	Fatal generalization	human	Eis-Hübinger et al. 1990 Lancet 336:880	HQ420896
CPXV_GER2002_MKY	Germany, Göttingen	2002	Fatal generalization	marmoset (*Callithrix jacchus*)	Mätz-Rensing et al. 2006 Vet Pathol 43:212	HQ420898
CPXV_FRA2001_NANCY	France, Nancy	2001	Local lesions	human		HQ420894
CPXV_UK2000_K2984	United Kingdom, Bristol	2000	Local lesions	cat		HQ420900
CPXV_AUS1999_867	Austria, Texing	1999	Local lesions	cat		HQ407377
CPXV_GER1980_EP4	Germany, Hameln	1980	Local lesions	elephant	Pilaski et al. 1986 Arch Virol 88: 135–142	HQ420895
CPXV_GER1998_2	Germany, Eckental	1998	Local lesions	human	Pfeffer et al. 1999 BMTW 112:334–338	HQ420897

Information is given for CPXV isolates used in the phylogenetic analysis, including place and date of isolation, clinical information, reference and GenBank accession number.

Phylogenetic analyses were conducted using MrBayes with the following settings: nst = 6, rates = invgamma, mcmc, 2 M generations, samplefreq = 1000, nchains = 4 and burnin = 1000. PAUP* [19] was used to calculate pairwise genetic distances (uncorrected *p*) for comparisons between taxa. Patristic distances (tree branch lengths between taxa) between isolates were calculated from the consensus tree using Patristic (http://www.bioinformatics.org/patristic/). For both methods, distances were averaged across taxa to produce a value at each node. A threshold value was calculated for both the patristic and genetic distance using the distances between *Taterapox virus* (TATV) and *Variola virus* (VARV). These values (VARV-TATV) represent the distance between two currently recognized and undeniably distinct OPV species. Comparisons were then made between the other OPV species, including the CPXV isolates, to determine which of these exceeded the VARV-TATV threshold value.

## Results

**Figure 1:**
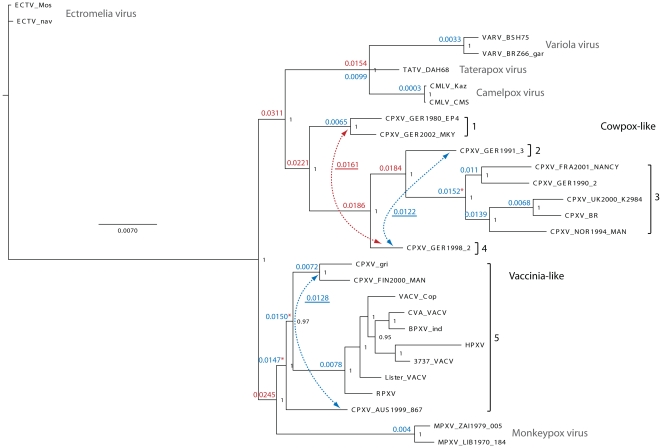
CPXV Phylogeny. The tree search was based on a complete coding region alignment (C23L-B29R) with gapped columns removed (145,177 bp). Posterior probabilities are shown to the right of each node in black. Uncorrected *p* distance measures were calculated from the matrix and are shown to the left of each node. Red indicates distances greater than or equal to the distance between TATV and the VARV clade (0.0154); blue indicates a distance less than this value. Additional distance measures between nodes and taxa are underlined and designated by dashed arrows. Asterisks indicate distance values at nodes where patristic distance values are above the VARV-TATV threshold and conflict with uncorrected *p* values. Cowpox-like (groups 1–4) and Vaccinia-like (group 5) clades are indicated to the right. Brackets encompass isolates or groups of isolates where distance measures separate them from their nearest neighbours at least as much as the distance between TATV and VARV. Each of these groups is labelled with a number (1–5).

**Figure 2:**
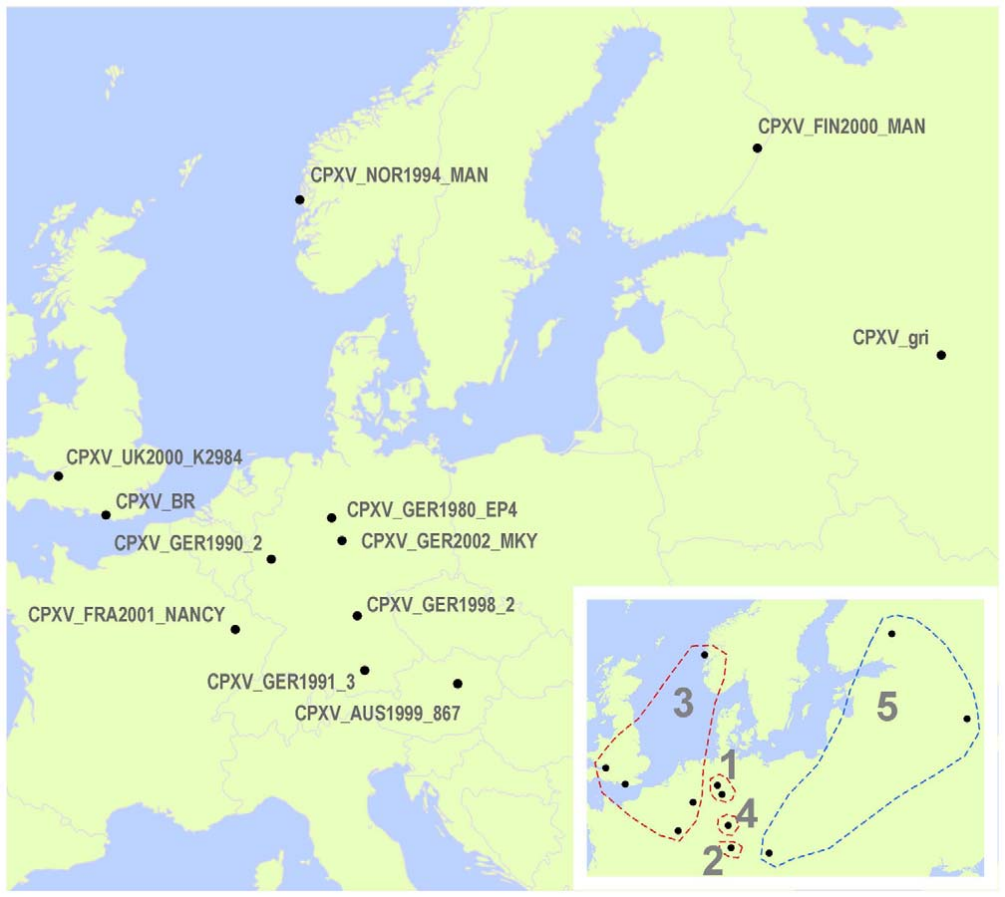
Map of CPXV isolate origins. The place of origin for each CPXV isolate is designated on the map. The inset shows CPXV isolates divided into five distinct clusters corresponding to those in Figure 1 and grouped by dashed lines.

The CPXV phylogenetic tree presented in Figure 1 depicts two major OPV clades. One includes three CPXV isolates most closely related to VACV (sister to MPXV) and the other is monophyletic (9 CPXV) and sister to VARV, TATV and CMLV. Using the TATV-VARV threshold (0.0154) as a guide, the CPXV isolates can be split into two major monophyletic clades (Cowpox-like and Vaccinia-like) and further into the five distinct monophyletic clusters. Distance measures for each of these clusters exceed the TATV-VARV threshold value. Table 1 has detailed information about the year and source of each isolate depicted on the tree and Figure 2 shows their geographic distribution. German CPXV isolates are found in 4 of the 5 groups (groups 1–4) which are all in the Cowpox-like clade and are the exclusive members of groups 1, 2, and 4. Group 1 is composed of two German isolates. Groups 2 and 3 are distinct sister taxa and are each represented by a single German CPXV isolate, and isolates from the UK, Norway, Germany, and France, respectively. Group 4 is a single German isolate which is sister to the 2–3 cluster. Group 5 is sister to a MPXV clade and forms the second major clade and consists of the Vaccinia-like isolates. This is the most speciose CPXV associated assemblage containing two separate monophyletic CPXV clusters as well as all of the included smallpox vaccine strains and other VACV isolates including Buffalopox virus (BPXV), Rabbitpox virus (RPXV) and Horsepox virus (HPXV). None of the genetic distances between isolates in group 5 exceeds the distance between TATV and VARV. When patristic distances are examined, three additional values exceed the threshold (marked with asterisks in Fig. 1) distinguishing a total of eight different clusters.

**Figure 3:**
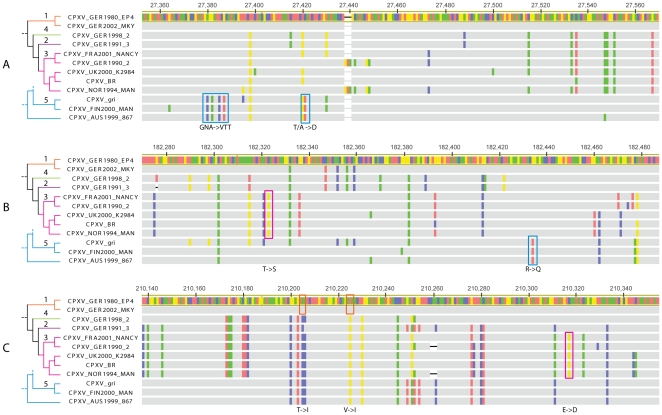
Alignment of three CPXV genes. Alignment images were created in Geneious Pro™ 5.3. Isolate names are provided to the right along with the branching relationships and numbered groups presented in Figure 1. Sequence areas depicted were extracted from the following coding regions: (A) CPXV_BR_021 (VACV_COP_C11R), secreted virulence factor, vaccinia growth factor (VGF; epidermal growth factor-like protein), promotes cell survival; (B) CPXV_BR_191 (VACV_COP_A53R), CrmC, viroceptor, soluble virus tumor necrosis factor receptor homolog, binds and antagonizes TNF-α; (C) CPXV_BR_212 (VACV_COP_B19R), viroceptor, soluble IFN α/β receptor homolog, binds and antagonizes IFN α/β. Numbers along the top of the alignment refer to the position of the region in the full genome alignment (supplementary material). Grey indicates conserved bases while colors (red, blue, yellow, green) indicate differences (A, C, G, T, respectively) with regard to the reference sequence (CPXV_GER1980_EP4) highlighted in yellow. Colored boxes identify shared nucleotide states that reflect monophyletic relationships seen in the tree. Notations below each alignment describe subsequent amino acid changes (standard IUPAC code).

To examine amino acid level properties of the identified CPXV clades, the coding sequence of three genes randomly selected from a list of relatively well characterized genes, (CrmC, viroceptor, soluble virus tumor necrosis factor receptor homolog [20]; soluble IFN α/β receptor homolog [21]; and vaccinia growth factor, epidermal growth factor-like protein [22]) linked to potential virulence factors were aligned for all CPXV isolates included in the phylogenetic analyses (Figure 3). Several unique non-synonymous mutations were found that unite each of the previously identified monophyletic lineages (groups 1, 3, and 5). In addition, group 3 is characterized by a truncation of gene C5L (unknown function). An image of the aligned CPXV genomes and their respective open reading frames is available in the online supporting material, Figure S1.

## Discussion

Although previous researchers have recognized that substantial genotypic and phenotypic diversity exists among CPXV species, to date no one has objectively analyzed this diversity and put it into a taxonomic context relevant to the other members of the Genus *Orthopoxvirus*. The data herein depicts CPXV as a polyphyletic assemblage in which one of the major CPXV clades is more closely related to MPXV and the other is more closely allied to variola, camel and tatera pox viruses. This would mean that if the current CPXV taxonomic scheme is to remain an intact monophyletic clade then, all *Orthopoxvirus* isolates with the exception of *Ectromelia virus* and the new world species, *Raccoonpox virus*, *Skunkpox virus* and *Volepox virus*, would need to be assigned to the species *Cowpox virus*. A more reasonable solution is to rename the distinct CPXV lineages.

The fact that the traditionally defined CPXV have the largest genomes has led previous investigators to suggest that a cowpox-like virus is the ancestor of all OPV species [23]. Furthermore, the deepest divergence within OPV clades is between the North American OPV species (Skunkpox, Racoonpox, and Volepox viruses) and the remainder of the genus, followed by *Ectromelia virus* [24]. The deep node from which the New World OPXV species diverge, along with the results herein suggest that the shared CPXV phenotypic characteristics such as; large genome size, A type inclusions, flattened hemorrhagic lesions on CAM, and rabbit lesion morphology, uniting the traditionally recognized CPXV isolates, likely represent ancestral characteristics maintained in the distinct CPXV lineages but lost by other OPV species.

The smallpox vaccine is known to have originated in the United Kingdom, however the vaccine strains were most closely allied to CPXV isolates from Russia (CPXV-GRI) and from Finland (CPXV_Fin2000_Man), with the Austrian isolate (CPXV_AUS1999_867) joining just outside that sister relationship. This could be explained by a lack of sufficient UK isolates in our analyses, which would imply that multiple CPXV and/or VACV genotypes are circulating in that region that have not yet been identified. The pairwise identity between the coding regions of CPXV_UK2000_K2984 and CPXV_BR is 98.4%. These two isolates were collected 63 years apart, but are both from the southern region of England and the Bristol isolate is roughly 23 miles from the region where Jenner's vaccination work occurred. Thus the genotypes represented in these analyses should be representative of the CPXV strains available to Jenner. A more likely scenario is that most of the commercially produced vaccines (all of those included here) were not made from the original Jenner strain, but instead from isolates found in other regions of Europe. Taxonomically, the UK isolates from near the “type” locality (i.e. near Jenner's original collection site) have priority to keep the *Cowpox virus* designation. In future studies, the inclusion of more sequences from other circulating vaccinia-like strains would be of evolutionary, epidemiological, and taxonomic importance.

The Buffalopox virus and Horsepox virus isolates are imbedded within the Vaccinia-like clade. There are multiple hypotheses as to the origin of *wild* VACV isolates [25]. The two prevailing scenarios are 1) these isolates represent vaccine derived strains that were utilized during the smallpox vaccination campaign and subsequently escaped and became endemic in these regions or 2) they are native viruses. The topology displayed here favors the first scenario (Figure 1). However, the long branch separating Horsepox virus from other VACV isolates suggests that more detailed analyses with additional sampling are needed to successfully resolve this discussion.

Both the historic and current literature describe “cowpox” as a disease with a single etiologic agent. Further, there has been confusion over the relationship between the causative agents of “cowpox” and “horsepox”, and the transition from what was historically called “cowpox” to the modern VACV-based vaccine strains. Much of this confusion results from the practice of naming agents after the host in which the resultant disease manifests. Using the TATV-VARV genetic distance threshold, CPXV isolates could be split into as many as five distinct monophyletic lineages. This threshold is likely conservative and is higher than the value seen between TATV and CMLV, two commonly accepted distinct OPXV species. If the lower TATV-CMLV threshold is used then Austrian, Norwegian, and British isolates are also distinct. This latter scenario would taxonomically differentiate the most geographically distinct isolates. The nucleotide data in Figure 1 and the coding data in Figure 3 support the elevation of up to four new species designations within the traditional CPXV group. According to taxonomic priority our clade 3 (Figures 1&2) would maintain the designation *Cowpox virus* since it contains isolates from the United Kingdom closest to the type locality from which the original CPXV isolates were described. If the taxonomic changes proposed herein are correct then it is likely that yet to be discovered biological similarities exist which unite the depicted clades/species. We chose to examine three genes that have been relatively well characterized in previous studies, for evidence of amino acid level changes which unite the proposed clades and found supporting data in all three loci. The nonsynonymous mutations seen in the coding sequence data for CrmC, viroceptor, soluble virus tumor necrosis factor receptor homolog [20]; soluble IFN α/β receptor homolog [21]; and vaccinia growth factor, epidermal growth factor-like protein [22] indicate that there are likely common protein structures shared within members of clades 1, 3 and 5 which could impact virulence and/or host range, thus supporting their recognition as distinct lineages. Furthermore, all members of clade 3 share a truncated C5L gene. We anticipate that future studies will reveal many other unique biological properties within each of the clades identified by this research effort.

In the most conservative view, our results show that CPXV strains group into at least two separate strongly supported and deeply divided clades. One clade includes VACV strains (both wild and laboratory propagated) while the other includes only strains previously identified as CPXV. This is congruent with previous observations differentiating CPXV Brighton Red and Grishak into two species [15]. Additional virological characterization is needed to specifically examine and subsequently assign these clades to the correct taxonomic category, however, all scenarios described herein suggest that a re-classification of CPXV strains is clearly warranted. Historic literature states that the original vaccine strains were derived from “cowpox” samples [2]. Additionally, the phylogeny presented here suggests the progenitor of the modern smallpox vaccine likely lies in mainland Europe and not the UK.

## Supporting Information

Figure S1 Image of twelve CPXV genomes created in Geneious Pro™ 5.3. The alignment was created using the MAFFT plugin. Orange arrows indicate coding regions in both directions. Bases are numbered at the top of the alignment and strain names indicated to the left. The reference genome, CPXV_GER1980_EP4, is highlighted in yellow. In subsequent genomes, grey indicates identity and vertical black lines represent differences with respect to the reference sequence.
(TIF)
